# A rare case of persistent visual aura in a 12 year old girl

**DOI:** 10.1186/1129-2377-14-S1-P1

**Published:** 2013-02-21

**Authors:** P Prabhakar, JC Simpson

**Affiliations:** 1 Great Ormond Street Hospital, UK

## Introduction

Migraine is a common neurological disorder affecting between 3% and 10% of children[1]. In up to 30% of sufferers [2], the headache is preceded or accompanied by a complex of neurological symptoms known as an aura. When aura symptoms persist beyond 7 days without evidence of infarction, the International Headache Society characterizes the condition as persistent migraine aura without infarction [3].

## Purpose/objectives

Persistent visual aura symptoms are rare, and only two published cases describe the condition in children [4,5]. We use this case to exemplify the condition and how it has been managed in our specialist clinic. We highlight the need for further insight to allow effective management in the paediatric population.

## Methods

The case of a 12 year old girl who has experienced persistent visual aura symptoms continuously since May 2010 is described, including the results of investigations and treatment history. The patient’s illustration and verbal description of symptoms provides a unique insight into her ordeal.

## Results

Our patient’s aura symptoms have so far been resistant to pharmacological therapy.

## Conclusion

Aura persistence in children lasting months is unusual. Our specialist clinic has only seen 2 previous cases, in which the aura lasted less than a week, and resolved with treatment for the migraine. As advances are made in our pathophysiological understanding, further treatment options may be discovered. Until a proven treatment is identified, it is important that clinicians share their experiences to help guide patient management.
